# Ensuring safety of DNA vaccines

**DOI:** 10.1186/1475-2859-4-26

**Published:** 2005-09-06

**Authors:** Jacob Glenting, Stephen Wessels

**Affiliations:** 1 Bioneer A/S, DK-2970 Hørsholm, Denmark; 2 Danish Toxicology Centre, DK-2970 Hørsholm, Denmark

## Abstract

In 1990 a new approach for vaccination was invented involving injection of plasmid DNA *in vivo*, which elicits an immune response to the encoded protein. DNA vaccination can overcome most disadvantages of conventional vaccine strategies and has potential for vaccines of the future. However, today 15 years on, a commercial product still has not reached the market. One possible explanation could be the technique's failure to induce an efficient immune response in humans, but safety may also be a fundamental issue. This review focuses on the safety of the genetic elements of DNA vaccines and on the safety of the microbial host for the production of plasmid DNA. We also propose candidates for the vaccine's genetic elements and for its microbial production host that can heighten the vaccine's safety and facilitate its entry to the market.

## Introduction

Vaccination with purified plasmid DNA involves injection of the plasmid into the patient to elicit an immune response to a protein that is encoded on the plasmid [1]. This mini-review focuses upon several aspects of safety of the DNA molecule itself and of the microorganism used to manufacture the DNA. The review is not exhaustive but does raise very important safety issues to be kept in mind early in the development of DNA vaccines.

DNA vaccination was described in a study in 1990 that demonstrated the induction of gene expression following direct intramuscular injection of plasmid DNA in mice [2]. Since then our understanding of the immunological mechanisms behind this unexpected result has increased. This includes identification of immune stimulatory DNA sequences (ISS) that could explain how DNA vaccines can evoke an immune response without an adjuvant [3]. The advantages of DNA vaccines over the traditional attenuated or subunit vaccines are their capacity to induce a broad spectrum of cellular and humoral immune responses, their flexible genetic design and low cost of production in a microbial host. Almost two thousand papers have been published, and several clinical trials have been conducted testing DNA vaccines against infectious diseases such as HIV-1 [4], Ebola virus [5] and malaria [6], or to generate protective immunity against tumors [7]. Despite this extensive research, a commercial product has yet to come to the market. One reason for this may be their failure to induce a strong immune response in higher animals like primates [8]. Another reason for their absence from the market may be related to their safety. Indeed, international regulatory groups have recently questioned the safety of certain existing DNA vaccine constructs and their production systems [9]. While the main focus of research has previously been on their functionality and immunological mechanisms, work on safety aspects most often is put off until later in development. By then, making fundamental changes to the DNA vaccine to improve its safety can be extremely costly and time-consuming.

In the following we propose some basic choices related to safety to be made during the development of DNA vaccines. We highlight safety issues that can be addressed by the appropriate choice of the vaccine's genetic elements, of its microbial production host and of the conditions of manufacture. Special focus will be put on the use of food-grade host-vector systems that are based on our experience with the lactic acid bacterium *Lactococcus lactis*.

### The vaccine's genetic elements

**Figure 1:**
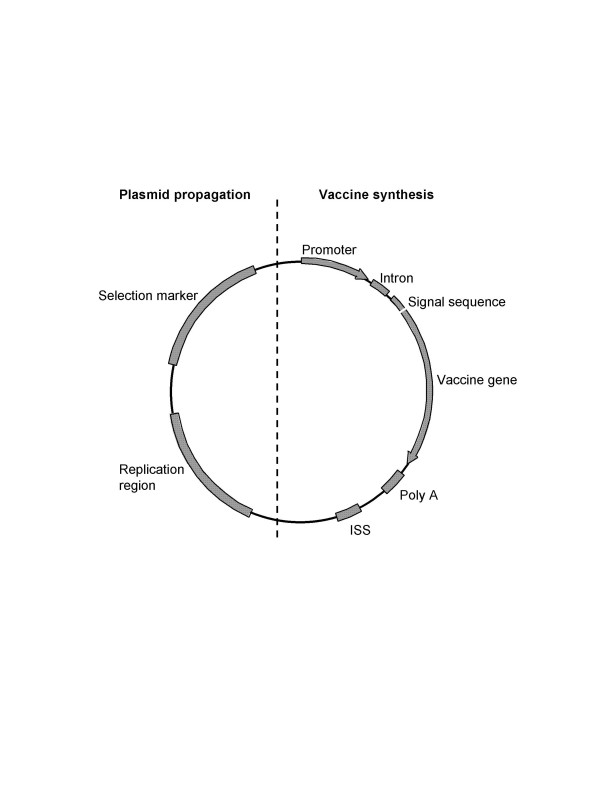
**Genetic elements of a plasmid DNA vaccine**. Plasmid DNA vaccines consists of a unit for propagation in the microbial host and a unit that drives vaccine synthesis in the eukaryotic cells. For plasmid DNA production a replication region and a selection marker are employed. The eukaryotic expression unit comprises an enhancer/promoter region, intron, signal sequence, vaccine gene and a transcriptional terminator (poly A). Immune stimulatory sequences (ISS) add adjuvanticity and may be localized in both units.

The organization of the genetic elements of a DNA vaccine reflects the plasmid's functionality, its bulk manufacture and its clinical use in the patient. Thus, the plasmid contains one unit responsible for its propagation in the microbial host and another unit that drives the expression of the vaccine gene in the cells of the patient. The genetic elements of the vaccine are shown in Figure 1, and particular safety concerns are listed in Table 1.

**Table 1:** The safety concerns and possible solutions for plasmid DNA vaccines and their production hosts. A priori each safety concern should be addressed as early in development as possible.

	Safety concern	Possible solution
Genetic elements	Transfer of plasmid to host flora	Narrow host-range replication region Non-antibiotic plasmid marker
	Germline integration	Avoidance of mammalian replication region
	Insertional mutagenesis and oncogenesis	Artificial DNA for promoter, intron, and signal sequence Avoidance of human-homologous DNA
	Adverse effects of encoded peptide(s)	Artificial signal sequences Avoidance of mammalian replication region Evaluation of vaccine peptide case-by-case
	Induction of autoimmune reactions	Minimized plasmids

Production host	Endotoxins and biogenic amines	Use of gram-positive organism
	Transferable antibiotic resistance genes	Determination of minimal inhibitory concentrations (MIC's) Screening for transferability
	Genetic instability	Analysis of plasmid population by sequencing and mass spectrometry
	Pathogenicity	Use of food-grade organism

The unit responsible for plasmid propagation in the microbial host contains a replication region and a selectable marker. The replication region allows the maintenance of multiple copies of the plasmid per host cell and a stable inheritance of the plasmid during bacterial growth. Furthermore, the replication region also determines the plasmid's host-range. Because DNA vaccination involves injection of milligram quantities of plasmid, replication regions with a narrow host-range can reduce the probability for spread of the plasmid to the patient's own flora. A replication region dependent on chromosomally encoded factors restricts the replication to a single host strain. One such bio-containment system has been developed in *E. coli *based on trans-complementation of a *rep*A^- ^plasmid replication region by a *rep*A^+ ^host strain [10]. Here, the pWV01-derived vectors cannot replicate in the absence of the replication factor RepA and thus relies on a *rep*A^+ ^helper strain. Addition of another *ori *(origin of replication) region that is active in mammalian cells allows prolonged persistence and expression of the vaccine gene in the transfected tissue. However, uncontrolled expression of the vaccine gene may induce immunological tolerance. Furthermore, persistence and increased spread of the plasmid may lead to germline transmission as a result of transfection of sperm cells or oocytes [11]. In fact, PCR studies have detected vaccine plasmid in the gonads of vaccinated fetuses and in offspring of these fetuses [12]. A literature study has identified non-replicating plasmids as a factor that reduces risk of germline transmission [13]. Accordingly, only prokaryotic and narrow host range replication regions should be present on vaccine plasmids.

Selectable markers ensure stable inheritance of plasmids during bacterial growth (Fig. 1). Most vaccine plasmids rely for this on resistance to antibiotics. Although a powerful selection, resistance genes to antibiotics are discouraged by regulatory authorities [14]. The concern is that the plasmid may transform the patient's microflora and spread the resistance genes (Table 1). Indeed, there is much international scientific and regulatory focus on this issue [15-19]. A non-antibiotic-based marker on vaccine plasmids for use in *E. coli *has been developed. This system is based on the displacement of repressor molecules from the chromosome to the plasmid, allowing expression of an essential gene [20]. A selection marker developed in our laboratory uses an auxotrophic marker in *L. lactis *[21,22]. Here, genes encoded on the plasmid relieve the host's threonine requirement. This selection system is efficient and precludes the use of antibiotics.

The nature of the DNA between the functional genes in vaccine plasmids is also a safety concern. Specific DNA sequences or methylation patterns can induce anti-DNA antibodies and lead to the autoimmune disease systemic lupus erythematosus [23]. Gilkeson et al. showed that amongst various organisms bacterial DNA induced the highest level of DNA-specific antibodies [24]. Therefore, a reasonable strategy is to minimize the non-functional sequences in the vaccine plasmid (Table 1). Vaccine plasmids have been developed which omit the prokaryotic backbone using an integrase-mediated recombination technology [25]. In addition, these mini-circles showed higher *in vivo *gene expression than a standard plasmid. Alternatively, we have used a plasmid backbone derived entirely from food-grade bacterial DNA [26].

The vaccine expression unit consists of the elements necessary for high-level expression and targeting of the vaccine component (Figure 1). Most DNA vaccines harbor promoters and enhancer regions from pathogenic viruses such as cytomegalo virus (CMV), simian virus 40, or murine leukaemia virus. For instance, plasmid vaccines with the CMV promoter have been in clinical trials and are versatile due to the promoter's activity in a variety of tissues and animal models [27]. As more than 50% of the population in USA is infected with CMV and as the virus remains in the body throughout life [28], the use of its expression signals on vaccine plasmids may induce recombination events and form new chimeras of CMV. Promoters and enhancer regions have also been suggested from housekeeping genes encoding the mouse phosphoenolpyruvate carboxykinase and phosphoglycerate kinase [29]. However, due to the risk of insertional mutagenesis and oncogenesis, highly inter-species-conserved sequences like these should be avoided. This risk can be reduced by the use of novel synthetic promoters selected by bioinformatic tools to have a low homology to sequences potentially present in the recipient. To augment the promoter activity, introns are introduced, which have a beneficial effect on the *in vivo *expression of the vaccine gene [30]. Most often the intron A from CMV is used. Here, too, bioinformatics can aid in the design of synthetic introns thereby avoiding sequences already present in CMV-infected individuals.

For secretion of the vaccine peptide to the extra-cellular milieu, a signal sequence is positioned in front of the vaccine gene. This codes for a signal peptide of about 20–40 amino acids, often derived from bovine proteins such as the plasminogen activator [31]. However, the fusion of bovine peptides to an immunogen may induce an immunological cross-reaction. Signal peptides can themselves induce protective immunity against a microbial pathogen when administered as a gene vaccine [32]. Apparently, to avoid undesired immune responses, the nature of the signal peptide should be considered (Table 1). Statistical methods like the hidden Markov model have been used to predict and generate artificial signal peptide sequences for use in human cells [33]. Such a strategy could be applied to DNA vaccine development to create more appropriate signal peptides.

To enhance the potency of a DNA vaccine, ISS's are added to the plasmid (Figure 1). These are nucleotide hexamers that interact with Toll-like receptors and add adjuvanticity [34]. The function of the ISS is independent of its location on the plasmid and may be present in the prokaryotic backbone. In fact, Klinman eliminated ISS from the plasmid backbone and could partially restore the immunogenicity of the plasmid by exogenously added ISS DNA [35]. Therefore, changing the vector backbone or editing plasmid components may influence the immune response due to deletion of the ISS. This, too, emphasizes the importance of the proper selection of expression vector early in vaccine development.

### The microbial host and production of bulk purified plasmid

The characteristics of the microbial host affect the quality of the purified DNA [36]. A number of safety concerns have been advanced concerning the microbial host. As explained in the following, these include production of toxins and biogenic amines, transferable antibiotic resistances, and genetic instability, including prophage-induced promiscuity and rearrangement of plasmid DNA (Table 1).

For reasons of efficiency, *E. coli *is usually chosen today as the production host, with its concomitant benefits and drawbacks. The benefits include a high DNA yield and well-established procedures for down-stream processing of the plasmid. However, as a gram-negative bacterium, *E. coli *contains highly immunogenic endotoxin, or lipopolysaccharides (LPS), in its outer membrane. Because of the net negative charge of both LPS and DNA, these molecules may be co-purified by the ion exchange principle used in the purification of plasmid DNA, although commercial kits do exist that can exclude LPS. On the other hand, the use of gram-positive hosts, none of which produce LPS, eliminate this dependency on the absolute efficiency of LPS-removing kits. Although not as efficient for plasmid production, *L. lactis*, as a gram-positive, produces neither endotoxin nor biogenic amines [37]. Assay for transferable antibiotic resistances in lactic acid bacteria is today a routine procedure; common *L. lactis *research strains are also genetically robust; and their prophages are of narrow host-range [38,39].

For large-scale plasmid production, often in about a thousand liters, the fermentation medium must sustain a high-level production of biomass and of plasmid DNA. At the same time the medium should be chemically defined and without components of animal origin that may contain viruses or prions [40]. Growth in a synthetic medium for many organisms results in low biomass and low plasmid yield. Indeed, switching microbial host to increase yield is complicated as it may lead to unexpected immunological results because of different DNA methylation patterns. Consequently, the production strain should be evaluated in synthetic media at an early point in development. Also here, *L. lactis *may be the host of choice due to its efficiency of growth in chemically defined media [41,42]. Finally, the genetic integrity of bulk purified plasmid molecules is today primarily monitored by sequence analysis. However, to reveal minor populations of molecules such as multimers or molecules with deletions and insertions, mass spectrometry should be considered [43].

## Conclusion

Plasmid DNA vaccines could be the next generation of vaccines. As yet, research has focused on building functional DNA vaccines. Therefore, focus on safety has been limited. In this review we have mentioned some safety issues to be addressed early in vaccine development. Using bioinformatic tools, safe eukaryotic expression signals can be devised in synthetic DNA sequences. Safety may also be heightened by non-antibiotic plasmid selection markers, plasmid replication functions with narrow host-ranges, and minimized plasmids. Using a bio-containment strategy will also increase the safety of the microbial production host, as will avoidance of toxic substances like endotoxins. Synthetic growth media should be considered early in development and will influence choice of production host. Indeed, it can be easier to address several of these safety concerns early in vaccine development by basing the strategy on food-grade bacteria and their DNA, such as *L. lactis *and its DNA. Finally, the very availability of safe host-vector systems will most probably facilitate the overall acceptance of DNA vaccines.

## Authors' contributions

The author(s) contributed equally to this work.
